# Analysis of long non-coding RNA expression profiles in pancreatic ductal adenocarcinoma

**DOI:** 10.1038/srep33535

**Published:** 2016-09-15

**Authors:** Xue-Liang Fu, De-Jun Liu, Ting-Ting Yan, Jian-Yu Yang, Min-Wei Yang, Jiao Li, Yan-Miao Huo, Wei Liu, Jun-Feng Zhang, Jie Hong, Rong Hua, Hao-Yan Chen, Yong-Wei Sun

**Affiliations:** 1 Department of Biliary-Pancreatic Surgery, Ren Ji Hospital, School of Medicine, Shanghai Jiao Tong University, 1630 Dongfang Road, Shanghai 200127, P.R. China; 2 State Key Laboratory for Oncogenes and Related Genes, Key Laboratory of Gastroenterology & Hepatology, Ministry of Health, Division of Gastroenterology and Hepatology, Ren Ji Hospital, School of Medicine, Shanghai Jiao Tong University, Shanghai cancer Institute, Shanghai Institute of Digestive Diseases, 145 Middle Shandong Road, Shanghai 200001, P.R. China

## Abstract

Pancreatic ductal adenocarcinoma (PDAC) remains one of the most aggressive and lethal malignancies. Long non-coding RNAs (lncRNAs) are a novel class of non-protein-coding transcripts that have been implicated in cancer biogenesis and prognosis. By repurposing microarray probes, we herein analysed the lncRNA expression profiles in two public PDAC microarray datasets and identified 34 dysregulated lncRNAs in PDAC. In addition, the expression of 6 selected lncRNAs was confirmed in Ren Ji cohort and pancreatic cell lines, and their association with 80 PDAC patients’ clinicopathological features and prognosis was investigated. Results indicated that AFAP1-AS1, UCA1 and ENSG00000218510 might be involved in PDAC progression and significantly associated with overall survival of PDAC. UCA1 and ENSG00000218510 expression status may serve as independent prognostic biomarkers for overall survival of PDAC. Gene set enrichment analysis (GSEA) analysis suggested that high AFAP1-AS1, UCA1 and low ENSG00000218510 expression were correlated with several tumorigenesis related pathways. Functional experiments demonstrated that AFAP1-AS1 and UCA1 were required for efficient invasion and/or proliferation promotion in PDAC cell lines, while ENSG00000218510 acted the opposite. Our findings provide novel information on lncRNAs expression profiles which might be beneficial to the precise diagnosis, subcategorization and ultimately, the individualized therapy of PDAC.

Pancreatic cancer remains one of the most aggressive and lethal malignancies with an extremely poor prognosis worldwide^1^. It is the fourth and seventh leading cause of cancer-related deaths in USA and China, respectively^2^,^3^, and is projected to rank the second by 2030 in USA^4^. Despite 50 years of research and therapeutic development, it has a dismal overall median survival of 6 months^5^ and 5-year survival rate of approximately 7% currently^2^. Among all pancreatic cancer cases, pancreatic ductal adenocarcinoma (PDAC) accounts for approximately 85% whose most effective therapy is surgery plus appropriate chemotherapeutic strategy, but only 15 to 20% of patients are eligible for surgical resection due to late diagnosis and early metastasis^6^. Hence, novel and effective strategies against this devastating disease are urgently needed. Better understanding of the genetic and molecular disorders of the disease is the key to early diagnosis, appropriate treatment and improved prognosis of patients with PDAC.

In recent years, integrative genomic studies have revealed that the vast majority of the human transcriptome is noncoding RNAs (ncRNAs), which are divided into short noncoding RNAs (<200 nucleotides: containing rRNAs, miRNAs, snRNAs, snoRNAs, siRNAs, and piRNAs) and long noncoding RNAs (lncRNAs, >200 nucleotides)^7^,^8^,^9^. They all have limited or no protein-coding capacity. miRNAs, which have been the most extensively investigated, are found to be dysregulated and involved in most human carcinogenesis and other disease processes^10^,^11^. More recently, the roles of lncRNAs have attracted considerable attention and exploded interest^9^. Accumulating evidence indicates that lncRNAs are frequently aberrantly expressed in cancers^8^,^9^,^13^,^14^, and some of them play a significant role in oncogenic or tumor-suppressive pathways and have similar diagnostic and prognostic power to that of mRNA and miRNA signatures^8^,^14^,^15^,^16^. LncRNAs are believed to be implicated in diverse biological, developmental and pathological processes and act through mechanisms such as chromatin reprogramming, cis or trans regulation at neighboring genes and post-transcriptional regulation of mRNA processing^12^,^17^,^18^. LncRNAs achieve these regulatory specificity through modularity, assembling diverse combinations of proteins and possibly RNA (eg, mRNA, miRNA) and DNA interactions^12^,^18^. For example, the well-known lncRNA HOTAIR is overexpressed in breast cancer where it binds polycomb repressive complex 2 (PRC2). This leads to silence of a portion of the HOXD locus, inducing altered histone H3K27 methylation and gene expression, which further enhances cancer invasiveness and metastasis^19^. HOTAIR is also increased in expression in many other types of cancers, including pancreatic cancer^20^. It was found that HOTAIR was a negative prognostic factor for pancreatic cancer patients and exhibited pro-oncogenic activity in both vitro and vivo bioassays^21^. Another lncRNA, the lncRNA-activated by TGF-b (lncRNA-ATB) could promote hepatocellular carcinoma (HCC) cell invasion by competitively binding the miR-200 family, upregulating ZEB1 and ZEB2, and then inducing EMT. And enforced lncRNA-ATB in HCC metastases was associated with poor prognosis^22^. Although a set of human lncRNAs have been identified, but lncRNA expression patterns and their characteristics in PDAC remain largely unexplored. Previous study demonstrated that lncRNA profiling could be obtained by mining the existing gene expression microarray data because a large amount of lncRNA-specific probes were fortuitously represented on the commonly used microarray platforms^23^,^24^,^25^,^26^, allowing the identification of a wealth of lncRNA profiling data in PDAC.

In this study, we aimed at conducting lncRNA expression profiles and identifying a series of differentially expressed lncRNAs in PDAC compared with adjacent nontumor pancreatic tissues by analyzing a cohort of previously published PDAC microarray datasets from the Gene Expression Omnibus (GEO). The identified lncRNAs specific to different tissue types were then verified in another independent dataset. After that, we selected the top 8 differentially expressed lncRNA probes (corresponding to 6 lncRNAs) and validated them in Ren Ji cohort and pancreatic cell lines by quantitative real-time PCR (qRT-PCR). We further investigated whether the 6 lncRNAs expression levels were associated with PDAC patients’ clinicopathological features and prognosis and explored their possible biological pathways and processes involved and cell biological functions. Our findings provide novel information on lncRNAs expression profiles which might be beneficial to the precise diagnosis, subcategorization (such as clinical phenotypes and molecular subtypes) and ultimately, the individualized therapy of PDAC.

## Results

### Characteristics of microarray datasets and clinical samples

**Figure 1:**
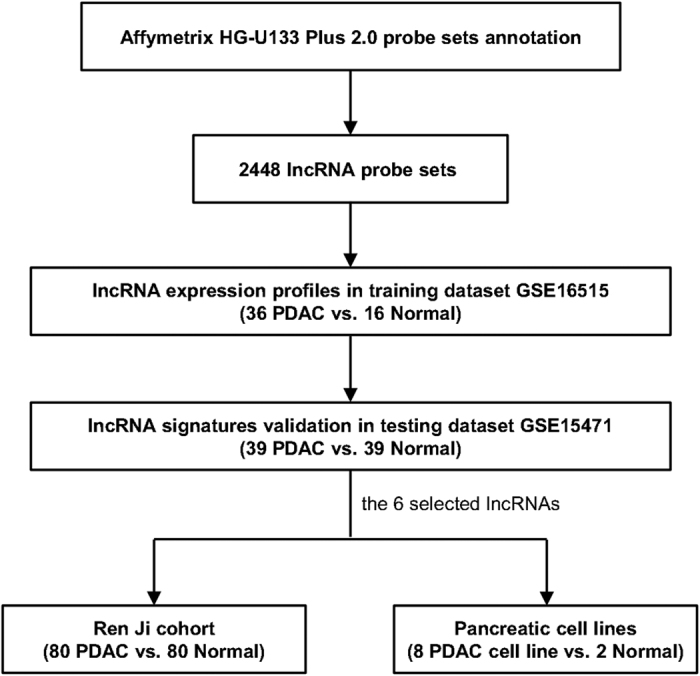
Flowchart of the study overview. LncRNA expression profiles were retrieved from Affymetrix HG-U133 Plus 2.0 microarray through a lncRNA-mining approach. LncRNA expression analyses were performed in the training dataset (GSE16515) first and then validated in the testing dataset (GSE15471). The 6 selected lncRNAs were then verified in Ren Ji cohort and pancreatic cell lines.

Our systematic study included two PDAC gene expression data from GEO: GSE16515 and GSE15471. GSE16515 consisted of 36 PDAC samples and 16 normal pancreatic tissue samples, a total of 52 samples. 16 pairs of samples consisted of both tumor and normal expression data, whereas the remaining 20 samples consisted of only tumor data. In GSE16515, there were 14 females and 22 males, with age ranging from 49 to 84 years old. GSE15471 contained 78 samples, including 39 PDAC tumors and 39 matching adjacent noncancerous tissue samples that were obtained from resected pancreas of 36 pancreatic cancer patients. 3 pairs were carried out replicate microarray hybridizations. Ren Ji cohort included 80 PDAC tumor samples paired with non-tumoral pancreatic tissue controls. Table 1 provides the clinical and histopathologic parameters for the 80 patients. While GSE16515 was used to derive a set of lncRNA expression signatures in PDAC, GSE15471 acted as the testing dataset to confirm the results. The selected distinctive lncRNA expressions were further to be verified in Ren Ji cohort and pancreatic cell lines. The work flow of the entire study designed is illustrated in Fig. 1.

### Distinctive lncRNA expressions between PDAC samples and normal pancreatic tissues

**Figure 2:**
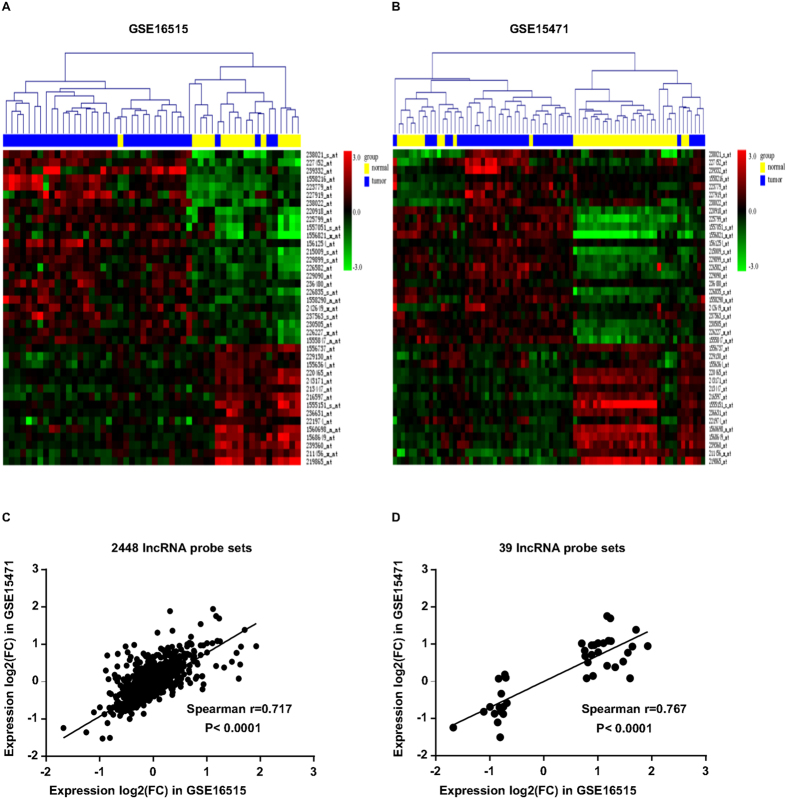
Distinctive lncRNA expressions between PDAC tissues and normal pancreas. (**A**) One-way hierarchical clustering of the 39 lncRNA probe sets (corresponding to 34 lncRNAs) identified as significantly different between PDAC tissues and normal pancreas in the training dataset (GSE16515). (**B**) Validation of the 39 probe signatures in the testing dataset (GSE15471). Each column represents one sample and each row represents one lncRNA probe set. Gene expression levels are indicated as follows: red, high expression (+3.0); green, low expression (−3.0). The bar colors in the dendrogram represent the sample types as indicated: blue, tumor; yellow, normal. (**C,D**) The distribution of all 2448 lncRNA probe sets (corresponding to 1970 lncRNAs) and the acquired 39 distinctive probe sets expression differentials between the experimental dataset GSE16515 and the validation dataset GSE15471.

Using the GSE16515 dataset as a training set for discovery and LIMMA with an adjusted P-value of less than 0.01 with a minimum fold change cut-off of 1.60 as a threshold, we first compared the lncRNA expression profiles of 16 normal pancreatic tissues to 36 PDAC tumors and identified 39 probe sets (corresponding to 34 lncRNAs) that were differentially expressed in PDAC (Table 2). Of these 39 deregulated probe sets, 24 probe sets (20 lncRNAs) were found to be up-regulated and 15 probe sets (14 lncRNAs) to be down-regulated. The top five most up-regulated lncRNAs (7 probe sets) in PDAC tumors were CRNDE, NR_036488, ENSG00000244649, AFAP1-AS1 and UCA1, while the top five most down-regulated lncRNAs (5 probe sets) were ENSG00000218510, ENSG00000244020, ENSG00000228536, ENSG00000251161 and ENSG00000236333. Nearly half of these lncRNAs don’t have an official Human Genome Nomenclature Committee (HGNC) symbol. The non-random partitioning of samples by unsupervised hierarchical clustering of these 39 probes (34 lncRNAs) clearly separated PDAC tissues from normal tissues (Fig. 2A). Only 6 samples (5 tumor samples and 1 normal samples) were misclassified by the clustering analysis. To independently confirm our results, we conducted the same analysis to identify the lncRNA signatures on the test dataset GSE15471 and found that 25 out of 34 differentially expressed lncRNAs identified by above analysis in GSE16515 also showed significant expression changes (adjusted P < 0.01, FC ≥ 1.60) with the same direction in GSE15471. And unsupervised hierarchical clustering of all samples in the GSE15471 dataset using the 39 lncRNA probe sets revealed similar distinctions between normal pancreatic tissues and PDAC samples: one group containing 28 of 39 non-tumoral samples and 5 PDAC samples, a second group containing 34 out of 39 PDAC samples and 11 normal samples (Fig. 2B). To further make this result convincing, we determined the correlation of the distribution of all 2448 probe sets (1970 lncRNAs) and the acquired 39 distinctive probe sets (34 lncRNAs) expression differentials between the experimental dataset GSE16515 and the validation dataset GSE15471. As shown in Fig. 2C,D, both of the two distribution of expression differentials was significantly concordant between the two datasets (r = 0.717, P < 0.0001; r = 0.767, P < 0.0001, respectively), suggesting a high consistence in expression patterns of these genes among different sample sets. Thus, the lncRNA signatures identified here were likely to be representative.

### Validation of candidate lncRNAs in PDAC patients and cell lines

**Figure 3:**
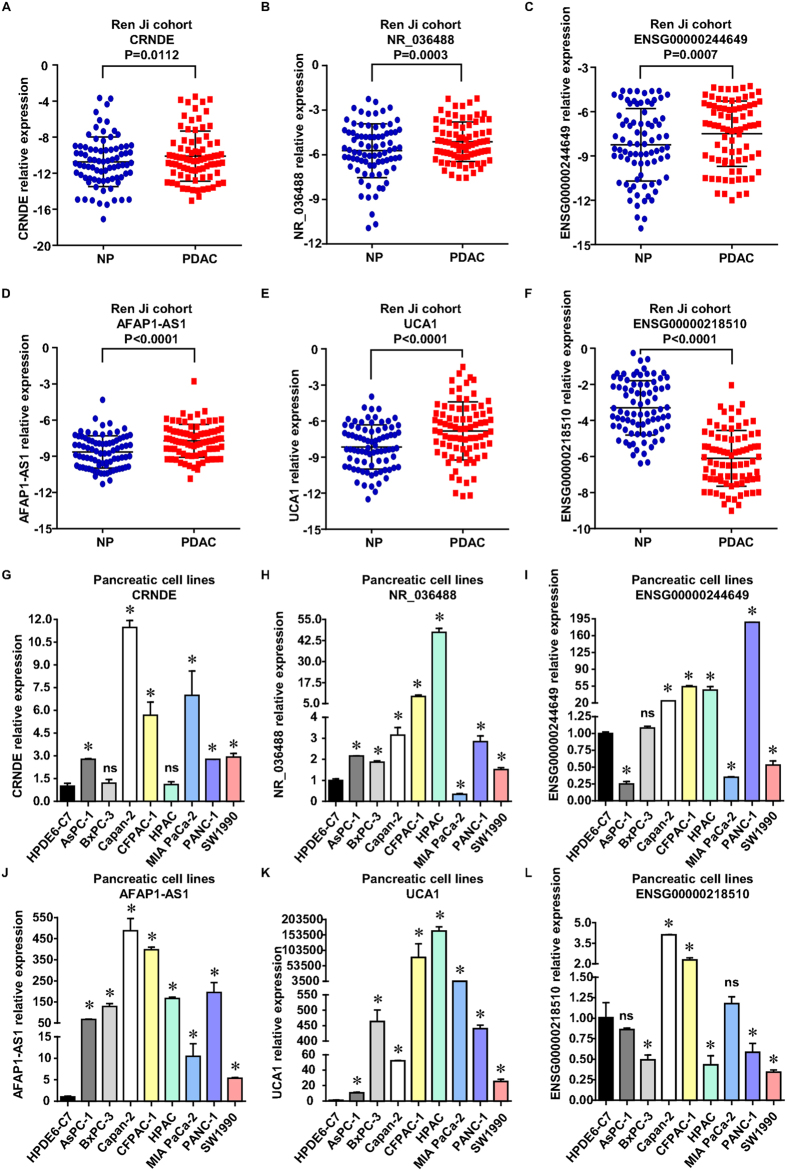
Validation of candidate lncRNAs in PDAC patients and cell lines by qRT-PCR analysis. (**A–F**) Analysis of CRNDE, NR_036488, ENSG00000244649, AFAP1-AS1, UCA1 and ENSG00000218510 relative expression in human PDAC and their matched normal pancreas samples, Ren Ji cohort, n = 80, paired sample t-test. (**G–L**) Relative expression level of CRNDE, NR_036488, ENSG00000244649, AFAP1-AS1, UCA1 and ENSG00000218510 in 8 PDAC cell lines compared to the nonmalignant HPDE6-C7 cell line. *P < 0.05 (Student’s t-test), ns: not significant. The qRT-PCR expression data were all shown as mean ± SD and normalised by GAPDH. GAPDH: glyceraldehyde-phosphate dehydrogenase; NP: normal pancreas.

To further corroborate the validity of our prior microarray findings, we selected the top 8 differentially expressed lncRNA probes (6 lncRNAs: CRNDE, NR_036488, ENSG00000244649, AFAP1-AS1, UCA1, ENSG00000218510) from the 39 deregulated probe sets between PDAC versus normal samples and analysed their expression by qRT-PCR in Ren Ji cohort and pancreatic cell lines. Ren Ji cohort included 80 PDAC samples and 80 matching adjacent normal tissue samples while cell lines contained 8 human PDAC cell lines and 2 normal pancreatic ductal epithelial cell lines. Of the 6 selected lncRNAs, 5 lncRNAs (CRNDE, NR_036488, ENSG00000244649, AFAP1-AS1, UCA1) were up-regulated and 1 lncRNA (ENSG00000218510) was down-regulated, and the fold change in mean expression intensity was ≥2.50 in GSE16515. The qRT-PCR results showed that CRNDE, NR_036488, ENSG00000244649, AFAP1-AS and UCA1 expression was significantly increased (P = 0.0112, P = 0.0003, P = 0.0007, P < 0.0001, P < 0.0001, respectively, Fig. 3A–E) and ENSG00000218510 expression was remarkably decreased (P < 0.0001, Fig. 3F) in PDAC tissues compared with paired normal pancreatic tissues. Similar result was also observed in PDAC and normal pancreatic ductal epithelial cell lines as demonstrated by the data (Fig. 3G–L and Supplementary Fig. S1A–F). Consistent with the findings in PDAC tissues, CRNDE, NR_036488, ENSG00000244649, AFAP1-AS1 and UCA1 expression was dramatically enhanced and ENSG00000218510 reduced in all or most of 8 PDAC cell lines in relative to the nonmalignant HPDE6-C7 cells or hTERT-HPNE cells at the RNA level. Our results thus highly coincided with our prior microarray findings in lncRNA expression profiles of PDAC.

### Relationship of the 6 selected lncRNAs expression with clinicopathological parameters and prognosis in PDAC patients

The relative expression of the 6 selected lncRNAs in PDAC tissues were categorized into low expression group (n = 40) and high expression group (n = 40) using the median value as cutoff. To explore the clinical significance of the 6 lncRNAs expression in PDAC, we analyzed the relationship between their expression and corresponding patients’ clinicopathological characteristics including age, gender, tumor location, TNM stage, tumor size, T classification, lymph node metastasis, distant metastasis, perineural invasion and histological differentiation in Ren Ji cohort. The results showed that in PDAC tissues AFAP1-AS1 was significantly associated with tumor size (≤3 cm vs. >3 cm; P = 0.044), UCA1 expression was significantly correlated with T classification (T1-2 vs. T3-4; P = 0.034), and ENSG00000218510 was prominently correlated with tumor size (≤3 cm vs. >3 cm; P = 0.044), distant metastasis (absent vs. present, P = 0.005) and histological differentiation (well/moderate vs. poor; P = 0.044), whereas no significant relevance was found with the other clinicopathological features and no significant associations were discovered between CRNDE, NR_036488, ENSG00000244649 expression and all of the clinicopathological parameters (Table 3).

**Figure 4:**
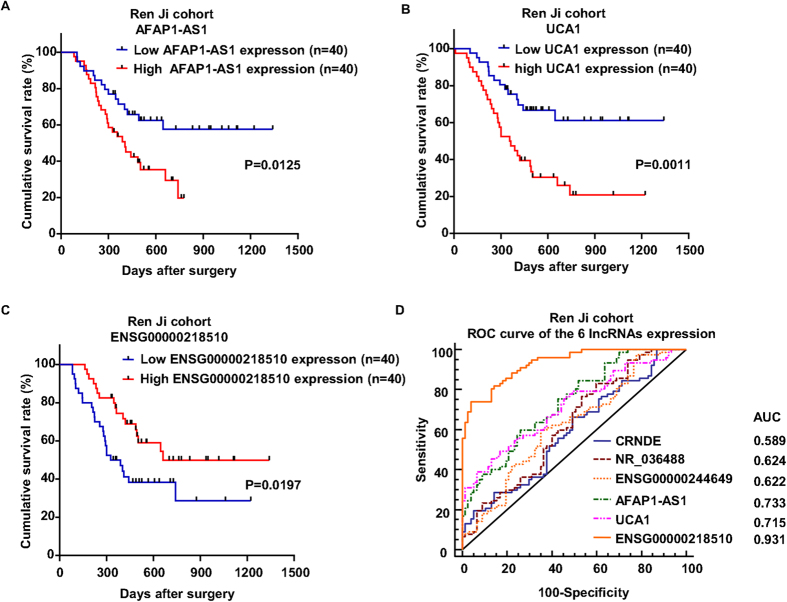
The potential value of candidate lncRNAs expression in predicting PDAC and patient prognosis. (**A–C**) Kaplan-Meier analysis of overall survival in Ren Ji cohort. Patients were scored as low and high expression group using the median value as cutoff according to lncRNAs expression. Results showed that patients with higher AFAP1-AS1, UCA1 and lower ENSG00000218510 expression have a poorer overall survival after surgery than their corresponding counterparts in PDAC. P-values were calculated by log-rank test. (**D**) ROC curve analyses of CRNDE, NR_036488, ENSG00000244649, AFAP1-AS1, UCA1 and ENSG00000218510 for prediction of PDAC using qRT-PCR-based expression level in Ren Ji cohort. AUC: the area under curve.

To further evaluate the prognostic significance of the 6 selected lncRNAs in PDAC patients, the correlations between their expression and corresponding clinical follow-up information were analyzed by Kaplan-Meier analysis and log-rank test. The results showed that AFAP1-AS1, UCA1 and ENSG00000218510 expression was remarkably associated with PDAC patients’ overall survival in Ren Ji cohort (P = 0.0125, P = 0.0011, P = 0.0197, respectively, Fig. 4A–C), which indicates that patients with higher AFAP1-AS1, UCA1 expression and lower ENSG00000218510 expression have significantly shorter survival time than those with lower AFAP1-AS1, UCA1 expression and higher ENSG00000218510 expression. The other 3 lncRNAs CRNDE, NR_036488 and ENSG00000244649 didn’t exhibit prognostic value in Ren Ji cohort (Figure not shown). Furthermore, univariable and multivariable analyses were performed to identify the risk factors correlated with patients’ prognosis. Univariable Cox regression analyses showed that tumor size (>3 cm vs. ≤3 cm), lymph node metastasis (present vs. absent), histological differentiation (poor vs. well/moderate), expression of AFAP1-AS1 (high vs. low), expression of UCA1 (high vs. low) and expression of ENSG00000218510 (high vs. low) were significant prognostic factors for overall survival prediction (Table 4). Meanwhile, multivariable Cox regression analysis displayed that lymph node metastasis (present vs. absent), expression of UCA1 (high vs. low) and expression of ENSG00000218510 (high vs. low) were independent predictors of PDAC patients’ overall survival after pancreatectomy (Table 4). Taken together, these data illustrated above suggested that part of the disregulated lncRNAs such as up-regulated AFAP1-AS1, UCA1 and down-regulated ENSG00000218510 could predict poor prognosis and might contribute to tumor progression in PDAC.

Furthermore, receiver operating characteristic (ROC) curve analyses were conducted to investigate the prediction of diagnostic value according to the 6 lncRNAs expression. Results demonstrated that the area under curve (AUC) of CRNDE, NR_036488, ENSG00000244649, AFAP1-AS1, UCA1 and ENSG00000218510 in Ren Ji cohort was 0.589, 0.624, 0.622, 0.733, 0.715, 0.931, respectively (Fig. 4D). These data indicated that part of the disregulated lncRNAs such as AFAP1-AS1, UCA1 and ENSG00000218510 may also be practical predictors for diagnosis in PDAC patients.

### Identification of lncRNAs associated biological pathways and processes

To gain further insight into the biological pathways and processes involved in PDAC pathogenesis through AFAP1-AS1, UCA1 and ENSG00000218510 pathway, Gene Set Enrichment Analysis (GSEA) was performed in tumor samples of GSE16515 datasets. NOM p-val < 0.05 and FDR q-val < 0.25 were considered as significant gene sets. Gene set differences in AFAP1-AS1 high vs low patients (median split) indicated that AFAP1-AS1 may regulate gene sets associated with I kappa B kinase NF-kappa B cascade, cell cycle phase and process, mitosis, mitotic cell cycle and so on (Supplementary Table S1A). High UCA1 expression was accompanied with up-regulation of gene sets associated with positive regulation of I kappa B kinase NF-kappa B cascade, regulation of growth, regulation of cell growth, cell migration, anti-apoptosis, growth and so on (Supplementary Table S1B). For ENSG00000218510, the gene signatures of cell cycle process and phase, mitosis, mitotic cell cycle, DNA replication, I kappa B kinase NF-kappa B cascade and so on were more correlated with patients with ENSG00000218510 lower expression versus patients with ENSG00000218510 higher expression (Supplementary Table S1C). These related pathways and processes were reported to be associated with tumorigenesis, and thus the lncRNAs signature might be involved with.

### Three signature lncRNAs regulate the proliferation and/or invasion abililty of PDAC cell lines

**Figure 5:**
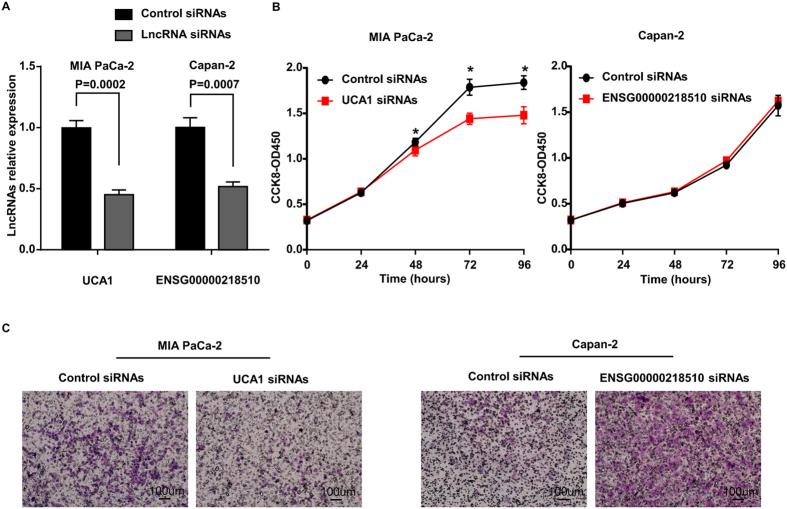
LncRNA UCA1 and ENSG00000218510 regulate the proliferation and/or invasion abililty of PDAC cell lines. (**A**) UCA1 and ENSG00000218510 knockdown efficiency was confirmed by qRT-PCR in MIA PaCa-2 and Capan-2 cell line, respectively. (**B**) The effect of UCA1 and ENSG00000218510 knockdown on PDAC cell line proliferation was determined by CCK-8 assays. *P < 0.05, Student’s t-test. Data are represented as the mean ± SD. (**C**) Representative images of transwell assay after UCA1 and ENSG00000218510 knockdown in MIA PaCa-2 and Capan-2, respectively.

The oncogenic function of AFAP1-AS1 has been reported in a recent study^27^. To evaluate the effects of UCA1 and ENSG00000218510 on cell biological behaviors, specific small interfering RNAs (siRNAs) were employed to knockdown UCA1 and ENSG00000218510 expression in human PDAC cell lines. According to their expression status in PDAC cell lines as described above, we selected the relative higher expression cell line MIA PaCa-2 for UCA1 experiment and Capan-2 for ENSG00000218510, respectively. The transfection efficiency was confirmed by qRT-PCR (Fig. 5A). Cell Counting Kit-8 (CCK-8) assays indicated that cell proliferation was decreased when UCA1 was knocked down, while ENSG00000218510 exhibited no difference (Fig. 5B). The results suggested that UCA1 played a physiological role in regulating cell proliferation. We further used Transwell assay to monitor the effect of manipulating UCA1 and ENSG00000218510 expression on cell invasiveness. Knockdown of UCA1 significantly reduced the number of PDAC cells that penetrated the Transwell filter, which demonstrated a substantial loss of cell invasion ability, while ENSG00000218510 the opposite (Fig. 5C).

## Discussion

Since the beginning of genomic technologies, many groups have tried to identify molecular biomarkers that would help elucidate the mechanisms of PDAC progression and malignant transformation^1^. LncRNAs, as a novel class of regulatory functional molecules involved in cancer biology, may yield valuable information and provide a different perspective^8^,^13^. In recent years, an increasing number of lncRNAs have been identified and associations between lncRNAs and various types of cancer have been investigated^8^,^9^. However, only a handful of studies have been reported in current PDAC cancer literature regarding lncRNA expression changes. LncRNA expression patterns and their characteristics in PDAC remain to be further systematically explored. Here, We investigated the lncRNA expression signatures in PDAC and evaluated their potential clinical and prognostic significance of several lncRNAs among them, hoping to shed more light on progression of the illness and of course, possible targets to develop new therapeutic drugs.

In this study, we profiled lncRNAs expression in PDAC tissues by mining two cohort of existing microarray gene expression datasets using Affymetrix HG-U133 Plus 2.0 array platform, since a great number of lncRNAs were interrogated on many commonly used commercial arrays. The robustness of this mining approach has been applied by several researchers and it is proved to be feasible, reliable and of low cost^24^,^25^,^26^. Based on this method, we identified a set of 34 lncRNAs that differentiated (adjusted P < 0.01, FC ≥ 1.60) between PDAC and normal pancreatic tissues. Such differentiation implied their potential roles in pancreatic tumorigenesis. Among the 34 dysregulated lncRNAs, some have been demonstrated to be related to PDAC. For example, AFAP1-AS1, which was significantly up-regulated (with an average fold change of about 3) in our data, has been reported to be overexpressed in PDAC and its overexpression was associated with lymph node metastasis, perineural invasion and poor survival^27^. AFAP1-AS1 suppression resulted in attenuated PDAC cell proliferation, migration, and invasion *in vitro* functional experiments, while ectopic expression the opposite. *In vivo* inhibition of AFAP1-AS1 impaired pancreatic cancer cell tumorigenicity. Our results also confirmed its aberrant expression and prognostic significance in PDAC, but in our study, AFAP1-AS1 expression was only found to be correlated with tumor size, its relation with lymph node metastasis and perineural invasion wasn’t significant, which may have been a consequence of the small sample size in Ren Ji cohort. Another candidate, lncRNA PVT1, was found to be up-regulated (with an average fold change of 1.84) in our study. This finding was in concordance with recent reports that increased expression of lncRNA PVT1 in PDAC was correlated with clinical stage and N-classification, and PVT1 might act as a potential molecular biomarker for predicting the prognosis of patients with PDAC^28^. Besides, PVT1 was also identified as a regulator of Gemcitabine sensitivity and functional inactivation of it led to enhanced Gemcitabine sensitivity in human pancreatic cancer cells^29^. More interestingly, recent researches identified several new risk loci significantly associated with susceptibility to pancreatic cancer through conducting genome-wide association study (GWAS), among which one locus is at 8q24.21 located 455 kb telomeric of PVT1^30^ and one at 17q25.1 (LINC00673)^31^. Coincidentally, PVT1 and NR_036488 (LINC00673) expression were both elevated in PDAC in our result. Whether there exist interactions between them remains to be investigated. In addition, we performed a comparsion between our results and the findings of a recent study^32^, which identified differential expression of 43 lncRNAs (FDR < 0.05, |log2FC| > 1.6) in six PDAC and five normal control tissues from 432 lncRNAs detected using massive analysis of cDNA ends (MACE). The contrast result showed that 6 elevated lncRNAs NR_036488 (LINC00673), AFAP1-AS1, NR_026812 (RUNX1-IT1), ENSG00000222041 (LINC00152), ENSG00000233429 (HOTAIRM1), PVT1 and 4 diminished lncRNAs ENSG00000218510 (LINC00339), ENSG00000236333 (TRHDE-AS1), NR_026887 (CEBPA-AS1), ENSG00000213373 (LINC00671) in our result were also in their list with the same trend. However, functional investigations of most differentially expressed lncRNAs identified in our study, so far, have not been implicated in PDAC. But a handsome amount of lncRNAs such as ENSG00000222041 (LINC00152)^33^, DLEU2^34^, NCRNA00275 (ZFAS1)^35^, NR_024284 (ZEB1-AS1)^36^, ENSG00000241684 (ADAMTS9-AS2)^37^ have been reported to be involved in other types of cancers. Whether these lncRNAs play important roles in the development and progression of PDAC deserves further clarification.

Furthermore, our own qRT-PCR data confirmed the up-regulation of CRNDE, NR_036488, ENSG00000244649, AFAP1-AS1, UCA1 and down-regulation of ENSG00000218510 in PDAC and the subsequent analysis of their correlations with clinicopathological features and prognosis also indicated that part of them such as up-regulated AFAP1-AS1, UCA1 and down-regulated ENSG00000218510 may contribute to tumor progression and predict unfavourable prognosis in PDAC patients. This was further supported by examination of associated molecular pathways by GSEA analysis of GSE16515 tumors showing that up-regulated AFAP1-AS1, UCA1 and down-regulated ENSG00000218510 were more likely to involve with cell cycle, proliferation, I kappa B kinase NF-kappa B cascade and so on. They were reported to be associated with tumorigenesis, and thus the lncRNAs signature might be involved with. And a recent study^27^ and our following cell functional experiments further confirmed that the three lncRNAs regulate the proliferation and/or invasion abililty of PDAC cell lines. In addition, we attempted to seek some information and evidences regarding their sequence similarity, motifs and subcellular localization by searching the previous studies or several biomedical websites and softwares. For example, through RegRNA 2.0^38^, an integrated web server for identifying functional RNA motifs and sites, we could predict their possible motifs and some motifs in the results (Supplementary Table S3) such as UCA1 transcriptional regulatory motifs c-Myb, C/EBP have also been reported and validated in human bladder cancer^39^,^40^. Past studies also indicated that UCA1 is located in the cytoplasm by *in situ* hybridization^41^. We are also interested in conducting more experimental investigations on these lncRNAs’ information in PDAC in the next work. Moreover, the ROC curve analyses suggested a possible diagnostic value of AFAP1-AS1, UCA1 and ENSG00000218510. However, this was based on a limited numbers of patients. Larger scale studies are needed to confirm our findings and the function as well as mechanism underlying the dysregulation of these cancer-associated lncRNAs deserves further studies. Among the 6 selected lncRNAs, the functional study of AFAP1-AS1 in PDAC has been reported as described above, whereas the other 5 lncRNAs’ function has not been implicated in PDAC currently. But there have been emerging evidence that some of them played an important role in many other human malignancies. For example, CRNDE has been characterized as a carcinogenesis promoter in colorectal cancer^42^, and glioma^43^. Earlier study with microarrays has also shown that depletion of CRNDE (lincIRX5) with shRNAs targeting exon-containing transcripts resulted in the alteration of a series of genes associated with tumorigenesis^44^. Another lncRNA UCA1 has also been demonstrated to participate and manifest a oncogenic function in multiple types of cancers such as bladder cancer^45^, colorectal cancer^46^, breast cancer^47^, esophageal squamous cell carcinoma^48^, non-small cell lung cancer^49^, hepatocellular carcinoma^50^, gastric cancer^51^ and so on. Also, lncRNA NR_036488 (LINC00673, LOC100499467) was found up-regulated in non-small cell lung cancer^25^,^52^ and glioma^24^ through qRT-PCR or microarray analysis. The relevant study on the other two lncRNAs ENSG00000244649 and ENSG00000218510 in cancer has not been found so far and the roles they play warrant further study.

As is known to us, identification of biomarkers closely correlated with disease progression is particularly meaningful. Cancer is initiated by a series of accumulative genetic and epigenetic alterations that affect normal cellular identity, growth and differentiation. Gene expression profiling offers a comprehensive molecular understanding of cancer that may grant insights into its pathophysiology and yield relevant information for subtype classification, staging, prognosis and therapeutic decision-making. Nowadays, increasing studies suggest that lncRNAs are promising biomarkers of cancer and have the potential to act such a role. One of the most prominent example is PCA3, a lncRNA highly expressed in prostate cancer^53^. The functions of lncRNAs are more likely to closely associated with their expression level as they do not encode proteins^26^. Thus lncRNAs may have higher specificity than protein-coding mRNAs and be more suitable to serve as prognostic and/or predictive markers for PDAC. Moreover, lncRNAs have the advantages of being detectable in the blood^8^ and urine^9^,^12^,^53^ of cancer patients by PCR methods. The distinctive expression patterns of lncRNAs in PDAC may potentially aid the development of biomarkers and, subsequently, the diagnosis of PDAC. Differential expression profiles of lncRNAs may also guide future development of novel therapies. Currently, therapies designed to target cancer-driving lncRNAs are also under intensive investigation^54^. To this end, rapid advances in oligonucleotide and nanoparticle technology create realistic optimism for delivering the RNA interference (RNAi)-mediated gene silencing technology to regulate lncRNA levels *in vivo*.

The limitations of this study should be acknowledged. First of all, HG-U133 Plus 2.0 arrays represent only portion of the possible lncRNAs present. Secondly, for many of the the signature lncRNAs, information regarding their possible functional roles and mechanisms is still limited. More experimental investigations on these lncRNAs are needed. Thirdly, the sample size of each dataset is relatively small. Our analysis may also ignore some lncRNAs that other groups have demonstrated to be involved in PDAC progression due to the different distributions of the patient populations in terms of age, gender and stage of PDAC. We are also interested in exploring which lncRNAs are differentially expressed in different stages of PDAC. Unfortunately, the supplementary files of both two PDAC datasets we adopted didn’t provide related information. Last but not least, PDAC is a heterogeneous malignancy notable for its profuse desmoplastic stroma comprised of activated fibroblasts, leukocytes, and extracellular matrix^55^, thus it is ambiguous which of the genomic alterations and expression changes are due to neoplastic ductal epithelial cells and which simply reflect the differences in cellular composition.

In summary, we have identified and validated multiple novel lncRNAs which differentially expressed in human PDAC. Our findings indicate the potential roles of lncRNAs in PDAC, and may provide useful information in PDAC diagnosis, classification, prognosis and therapeutic evaluation. Future studies will focus on the verifications of identified lncRNA signatures and the functional elucidation of these lncRNAs.

## Materials and Methods

The methods were carried out in accordance with the approved guidelines.

The study was approved by the Research Ethics Committee of Ren Ji Hospital, School of Medicine, Shanghai Jiao Tong University and was performed in agreement with the approved ethical standards laid down in the 1964 declaration of Helsinki and all subsequent revisions.

### PDAC datasets preparation

PDAC microarray datasets and corresponding clinical data in this study were directly downloaded from GEO database. These datasets corresponded to all available public datasets fulfilling the following criteria: (i) they used PDAC tissue and normal pancreatic tissue for comparison; (ii) they used the same chip platform (Affymetrix HG-U133 Plus 2.0 array); (iii) they contained more than three samples meeting the quality control standard in experimental and controlled group. Two panels of PDAC gene expression datasets were included in our study: GSE16515 and GSE15471. We adopted the training-validation method. While GSE16515 was first used to identify the gene expression signatures, GSE15471 served as a validation dataset to evaluate the proportion of misclassifications.

### LncRNA profile mining

LncRNA expression profiles on Affymetrix GeneChip Human Genome HG-U133 Plus 2.0 array were pointed out in the light of the NetAffx annotation of the probe sets and the Refseq and Ensembl annotations of lncRNAs as described previously by Zhang^24^. To sum up, all transcripts represented on the microarray were first identified as protein-coding RNAs or ncRNAs, of which only the ncRNAs were retained and further screened by discarding pseudogenes, rRNAs, tRNAs, microRNAs and other short ncRNAs. Ultimately, total 2448 Affymetrix probe sets (corresponding to 1970 lncRNAs genes) were included in our following analysis. Among them, 725 probe sets (510 genes) mapping to lncRNAs were annotated by both the Refseq and the Ensembl databases; 512 probe sets (379 genes) were annotated only by the Refseq database, and 1211 probe sets (1081 genes) were annotated only by the Ensembl database. Those probe sets with controversial definitions in the two databases were excluded.

### Microarray data processing and analysis

The expression data of raw CEL files were normalized, log2 transformed and background adjusted utilizing a Bioconductor package Robust MultiArray Average (RMA)^56^ through R 3.2.0 software. After that, a set of probe ID-centric gene expression values were retrieved for downstream analysis. The normalized data were then analyzed with linear models for microarray data (LIMMA), a modified t-test incorporating the Benjamini–Hochberg multiple hypotheses correction technique^57^. The probe sets of which the adjusted P-value was below 0.01 and the expression level differed by ≥1.60-fold between two comparsion groups were characterized as significantly different lncRNAs.

Hierarchical clustering analysis (HCA) of the lncRNA profiles was performed using MeV 4.9.0 software^58^. The normalized expression values of the lncRNAs were centred on the median before performing unsupervised hierarchical clustering. Clustering was done with complete linkage and Euclidean distance. HCA was used to visually inspect the result.

GSEA was performed by the JAVA program (http://software.broadinstitute.org/gsea/index.jsp) using the GO gene sets database (c5.all.v5.1.symbols.gmt [gene ontology]) from the Molecular Signatures Database–MsigDB. The patients were stratified by the median of AFAP1-AS1, UCA1 and ENSG00000218510 expression level and significantly enriched biological pathways between classes under comparison were identified, which produced a nominal P-value of 0.05 and FDR of 0.25.

### Patients and samples

A total of 80 freshly-frozen primary pancreatic cancer and matched adjacent non-tumor tissues (Ren Ji cohort) were collected from patients who underwent pancreatic surgical resection at Ren Ji Hospital, School of Medicine, Shanghai Jiao Tong University between January 2012 and November 2014, and the pathological information was retrieved from the Pathology Department. None of the patients had received radiotherapy, chemotherapy, hormone therapy or other related anti-tumor therapies before surgery. For all samples from 80 patients, clinical information was available. The follow-up time was calculated from the date of surgery to pancreatic cancer-related death, or November 17, 2015, the ultimate deadline. All the patients were provided with written informed consent before enrolment, and the study was approved by the Research Ethics Committee of Ren Ji Hospital, School of Medicine, Shanghai Jiao Tong University.

### Cell culture and treatment

Human PDAC cell lines AsPC-1, BxPC-3, Capan-2, CFPAC-1, HPAC, MIA PaCa-2, PANC-1 and SW1990 were purchased from the Cell Resource Center, Shanghai Institute of Biochemistry and Cell Biology at the Chinese Academy of Sciences (Shanghai, China) and two immortalized normal human pancreatic ductal epithelial cell lines HPDE6-C7 and hTERT-HPNE were obtained from Professor ZG Zhang (Shanghai Cancer Institute, Shanghai, China). Cells were maintained in indicated medium according to ATCC protocols, supplemented with 10% (v/v) fetal bovine serum (FBS) and 1% antibiotics (100 μg/ml streptomycin and 100 U/ml penicillin) at 37 °C in a humidified incubator with a 5% CO_2_ atmosphere.

The siRNAs targeting human UCA1 and ENSG00000218510 were transfected into the PDAC cells using the DharmaFECT 1 siRNA transfection reagent (Thermo Scientific Dharmacon lnc.), whereas nonspecific siRNA acted as negative controls. The siRNAs were purchased from Genepharm Technologies (Shanghai, China) and sequences are listed in Supplementary Table S2A.

### RNA extraction and qRT-PCR

Total RNA was extracted from tissues and cells by using Trizol reagent (Takara, Shiga, Japan) and cDNA was synthesized using a PrimeScript RT Reagent Kit (Perfect Real Time; Takara, Shiga, Japan) according to the manufactuer’s instruction. StepOnePlus Real-Time PCR System (Applied Biosystems, Grand Island, NY, USA) was applied to detect the expression level of target gene using the SYBR Premix Ex Taq II (Takara, Japan), and the amplified transcript level of each specific gene was normalized to that of GAPDH. The fold change of target lncRNA’s expression intensity was calculated by the 2^−ΔΔCt^ method. Primer sequences are listed in Supplementary Table S2B.

### Cell proliferation assay

Cells were seeded into in 96-well plates at an initial density of 3000 cells/well and treated with 10 μl of CCK-8 (Dojindo Molecular Technologies, Kyushu, Japan) each well for 1.5 hours at the time points of 24 h, 48 h, 72 h and 96 h after the planting. Cell absorbance was detected by scanning with a microplate reader at 450 nm.

### Transwell chamber assay

After the chamber was coated with fresh Matrigel (diluted in 1:4 with serum-free medium) (BD Bioscience San Jose, CA, USA) in 24-well dishes, 2 × 10^5^ cells transfected with lncRNA siRNAs or control siRNA and suspended in serum-free medium were plated on the top of each chamber, while medium containing 20% FBS was placed in the lower chamber and used as a chemo-attractant. After 48 h incubation, cells that did not pass through the filter were removed by a cotton swab, whereas cells on the lower surface of the filter were fixed and stained with formaldehyde and crystal violet, respectively. The cells on the lower side of the filters were defined as invasive cells.

### Statistical analysis

Data were presented as the means ± SD. SPSS 22.0 software (IBM Corp., Armonk, NY, USA) was used for statistical analysis. Graphical representations were carried out with GraphPad Prism 6 (San Diego, CA, USA) and Medcalc 11.5 (Ostend, Belgium) software. For clinicopathological analysis, the chi-square test or Fisher’s exact test was performed. The survival calculations were illustrated with Kaplan-Meier curves and differences between survival curves were tested by the log-rank test. Cox proportional hazards model was used to identify the prognostic factors by univariable and multivariable analysis. The student’s t-test or Mann–Whitney U test was used for comparison between two groups depending on distribution. P values (two-sided) less than 0.05 were considered statistically significant.

## Additional Information

**How to cite this article**: Fu, X.-L. *et al.* Analysis of long non-coding RNA expression profiles in pancreatic ductal adenocarcinoma. *Sci. Rep.* **6**, 33535; doi: 10.1038/srep33535 (2016).

## Supplementary Material

Supplementary Information

Supplementary Table S1

Supplementary Table S3

## Figures and Tables

**Table 1:** Clinicopathological features of 80 patients with pancreatic ductal adenocarcinoma (PDAC).

Clinicopathological feature	N (%) of patients
Age (years)	
Median (range)	65 (37–91)
≤65	37 (46.25%)
>65	43 (53.75%)
Gender	
Male	45 (56.25%)
Female	35 (43.75%)
Tumor location	
Head	53 (66.25%)
Body/tail	27 (33.75%)
TNM stage	
Stage I	12 (15.00%)
Stage II	52 (65.00%)
Stage III	8 (10.00%)
Stage IV	8 (10.00%)
Size	
≤3cm	37 (46.25%)
>3cm	43 (53.75%)
T classificattion	
T1	2 (2.50%)
T2	11 (13.75%)
T3	57 (71.25%)
T4	10 (12.50%)
Lymph node metastasis	
Absent	46 (57.50%)
Present	34 (42.50%)
Distant metastasis	
Absent	72 (90.00%)
Present	8 (10.00%)
Perineural invasion	
Absent	39 (45.00%)
Present	41 (55.00%)
Histological differentiation	
Well/Moderate	43 (53.75%)
Poor	37 (46.25%)
CA199 level (U/ml)	
≤35	14 (17.50%)
>35	61 (76.25%)
Missing	5 (6.25%)
Survival status	
Median OS (Days)	493
Dead	43 (53.75%)
Alive	37 (46.25%)

OS: Overall survival.

Patients were staged in accordance with the 7th Edition of the AJCC Cancer’s TNM Classification.

**Table 2:** lncRNAs differentially expressed between PDAC samples and normal pancreatic tissues.

Probe set ID	Symbol	Fold change	Adjusted P value	HGNC symbol	Description
238021_s_at	CRNDE	3.80	1.78E-05	CRNDE	colorectal neoplasia differentially expressed (non-protein coding)
227452_at	NR_036488	3.26	1.59E-06	LINC00673	long intergenic non-protein coding RNA 673
239332_at	ENSG00000244649	3.12	1.90E-03		Uncharacterized LOC100506373
1558216_at	AFAP1-AS1	3.03	1.77E-04	AFAP1-AS1	AFAP1 antisense RNA 1
223779_at	AFAP1-AS1	2.94	5.89E-04	AFAP1-AS1	AFAP1 antisense RNA 1
227919_at	UCA1	2.77	2.65E-03	UCA1	urothelial cancer associated 1 (non-protein coding)
238022_at	CRNDE	2.50	6.67E-05	CRNDE	colorectal neoplasia differentially expressed (non-protein coding)
220918_at	NR_026812	2.37	2.56E-04	RUNX1-IT1	RUNX1 intronic transcript 1
225799_at	ENSG00000222041	2.35	7.10E-04	LINC00152	long intergenic non-protein coding RNA 152
1557051_s_at	ENSG00000233429	2.30	5.35E-03	HOTAIRM1	HOXA transcript antisense RNA, myeloid-specific 1
1556821_x_at	DLEU2	2.26	7.72E-03	DLEU2	deleted in lymphocytic leukemia 2 (non-protein coding)
1561254_at	ENSG00000228742	2.26	3.52E-04		
215009_s_at	ENSG00000251022	2.16	5.16E-05	THAP9-AS1	THAP9 antisense RNA 1
229899_s_at	NCRNA00275	2.01	1.36E-04	ZFAS1	ZNFX1 antisense RNA 1
226582_at	ENSG00000250742	2.01	1.44E-03		uncharacterized LOC400043
229090_at	NR_024284	1.91	5.88E-04	ZEB1-AS1	ZEB1 antisense RNA 1
236480_at	ENSG00000247095	1.88	7.40E-04	MIR210HG	MIR210 host gene
226835_s_at	NCRNA00275	1.85	8.20E-04	ZFAS1	ZNFX1 antisense RNA 1
1558290_a_at	PVT1	1.84	4.15E-03	PVT1	Pvt1 oncogene (non-protein coding)
242649_x_at	ENSG00000179362	1.76	1.90E-03		high mobility group nucleosomal binding domain 2 pseudogene 46
237563_s_at	ENSG00000233461	1.73	5.55E-03		
230505_at	NR_027046	1.70	6.86E-03		uncharacterized LOC145474
226227_x_at	NCRNA00275	1.69	8.52E-04	ZFAS1	ZNFX1 antisense RNA 1
1555847_a_at	NR_036515	1.63	4.41E-03		uncharacterized LOC284454
219865_at	ENSG00000218510	0.31	9.75E-05	LINC00339	long intergenic non-protein coding RNA 339
211456_x_at	ENSG00000244020	0.46	7.28E-06		metallothionein 1 pseudogene 2
239360_at	ENSG00000228536	0.50	1.33E-03		
1568649_at	ENSG00000251161	0.53	3.62E-03		
1560698_a_at	ENSG00000236333	0.55	4.65E-03	TRHDE-AS1	TRHDE antisense RNA 1
221974_at	IPW	0.56	2.42E-03	IPW	imprinted in Prader-Willi syndrome (non-protein coding)
1555151_s_at	ENSG00000154316	0.57	7.25E-04		L-threonine dehydrogenase
236631_at	ENSG00000188660	0.57	6.14E-03	LINC00319	long intergenic non-protein coding RNA 319
213447_at	IPW	0.58	5.82E-03	IPW	imprinted in Prader-Willi syndrome (non-protein coding)
216597_at	ENSG00000242115	0.58	6.67E-05		
220465_at	NR_026887	0.60	1.46E-03	CEBPA-AS1	CEBPA antisense RNA 1 (head to head)
243171_at	ENSG00000229196	0.60	1.36E-04		
229130_at	ENSG00000251615	0.61	1.44E-03		
1556364_at	ENSG00000241684	0.61	2.63E-03	ADAMTS9-AS2	ADAMTS9 antisense RNA 2
1556737_at	ENSG00000213373	0.62	2.25E-03	LINC00671	long intergenic non-protein coding RNA 671

HGNC: Human Genome Nomenclature Committee.

**Table 3:** Correlations between the six selected lncRNAs expression and clinicopathological features in patients with pancreatic ductal adenocarcinoma (PDAC).

Clinicopathological feature	CRNDE			NR_036488			ENSG00000244649			AFAP1-AS1			UCA1			ENSG00000218510		
	Low	High	P value	Low	High	P value	Low	High	P value	Low	High	P value	Low	High	P value	Low	High	P value
Age (years)																		
≤65	22	15	0.116	21	16	0.262	20	17	0.501	22	15	0.116	21	16	0.262	17	20	0.501
>65	18	25		19	24		20	23		18	25		19	24		23	20	
Gender																		
Male	22	23	0.822	23	22	0.822	25	20	0.260	20	25	0.260	25	20	0.260	21	24	0.499
Female	18	17		17	18		15	20		20	15		15	20		19	16	
Tumor location																		
Head	25	28	0.478	29	24	0.237	27	26	0.813	26	27	0.813	27	26	0.813	25	28	0.478
Body/tail	15	12		11	16		13	14		14	13		13	14		15	12	
TNM stage																		
Stage I+IIa	23	19	0.370	19	23	0.370	23	19	0.370	22	20	0.654	21	21	1.000	22	20	0.654
Stage IIb+III+IV	17	21		21	17		17	21		18	20		19	19		18	20	
Size																		
≤3cm	20	17	0.501	21	16	0.262	17	20	0.501	23	14	**0.044**	18	19	0.823	14	23	**0.044**
>3cm	20	23		19	24		23	20		17	26		22	21		26	17	
T classificattion																		
T1, 2	7	6	0.762	5	8	0.363	8	5	0.363	6	7	0.762	10	3	**0.034**	7	6	0.762
T3, 4	33	34		35	32		32	35		34	33		30	37		33	34	
Lymph node metastasis																		
Absent	24	22	0.651	21	25	0.366	24	22	0.651	24	22	0.651	23	23	1.000	25	21	0.366
Present	16	18		19	15		16	18		16	18		17	17		15	19	
Distant metastasis																		
Absent	36	36	1.000	35	37	0.456	36	36	1.000	35	37	0.456	36	36	1.000	32	40	**0.005**
Present	4	4		5	3		4	4		5	3		4	4		8	0	
Perineural invasion																		
Absent	18	21	0.502	20	19	0.823	17	22	0.263	18	21	0.502	20	19	0.823	18	21	0.502
Present	22	19		20	21		23	18		22	19		20	21		22	19	
Histological differentiation																		
Well/Moderate	20	23	0.501	11	11	1.000	18	25	0.116	22	21	0.823	19	24	0.262	17	26	**0.044**
Poor	20	17		9	9		22	15		18	19		21	16		23	14	

P values are calculated by χ^2^ test or Fisher’s exact test. The bold number represents the P-values with significant differences.

Patients were staged in accordance with the 7th Edition of the AJCC Cancer’s TNM Classification.

**Table 4:** Univariable and multivariable analyses of prognostic parameters for survival in patients with pancreatic ductal adenocarcinoma (PDAC).

Prognostic parameter	Univariable analysis			Multivariable analysis		
	HR	95% CI	P value	HR	95% CI	P value
Age (>65 vs. ≤65)	1.118	0.618–2.024	0.712	—	—	—
Gender (male vs. female)	1.196	0.661–2.163	0.554	—	—	—
Tumor location (head vs. body/tail)	1.053	0.568–1.953	0.870	—	—	—
Tumor Size (>3 cm vs. ≤3 cm)	1.954	1.054–3.623	**0.033**	1.252	0.649–2.417	0.503
T classification (T3, 4 vs. T1, 2)	2.142	0.765–6.001	0.147	—	—	—
Lymph node metastasis (present vs. absent)	2.352	1.292–4.284	**0.005**	1.906	1.006–3.609	**0.048**
Distant metastasis (present vs. absent)	1.240	0.551–2.787	0.603	—	—	—
Perineural invasion (present vs. absent)	1.277	0.702–2.326	0.423	—	—	—
Histological differentiation (poor vs. well/moderate)	2.240	1.221–4.109	**0.009**	1.586	0.816–3.084	0.174
Expression of CRNDE (high vs. low)	1.669	0.905–3.077	0.101	—	—	—
Expression of NR_036488 (high vs. low)	1.053	0.583–1.904	0.863	—	—	—
Expression of ENSG00000244649 (high vs. low)	1.027	0.568–1.856	0.929	—	—	—
Expression of AFAP1-AS1 (high vs. low)	2.205	1.167–4.169	**0.015**	1.678	0.851–3.310	0.135
Expression of UCA1 (high vs. low)	2.777	1.465–5.261	**0.002**	2.018	1.016–4.007	**0.045**
Expression of ENSG00000218510 (high vs. low)	0.486	0.262–0.903	**0.022**	0.493	0.254–0.957	**0.037**

HR: Hazard ratio; CI: Confidence interval. The bold number represents the P-values with significant differences.
