# Supplementary material for: Analysis of long non-coding RNA expression profiles in pancreatic ductal adenocarcinoma
Source: Sci Rep. 2016 Sep 15;6:33535. doi: 10.1038/srep33535 (PMC5024322; doi:10.1038/srep33535)
Supplement: Supplementary Information [file srep33535-s1.pdf]

Analysis of long non-coding RNA expression profiles in pancreatic ductal adenocarcinoma

Xue-Liang Fu[1,†], De-Jun Liu[1,†], Ting-Ting Yan[2,†], Jian-Yu Yang[1], Min-Wei Yang[1], Jiao Li[1], Yan-Miao Huo[1], Wei Liu[1], Jun-Feng Zhang[1], Jie Hong[2], Rong Hua[1,*], Hao-Yan Chen[2,*], Yong-Wei Sun[1,*]

Supplementary Figure S1

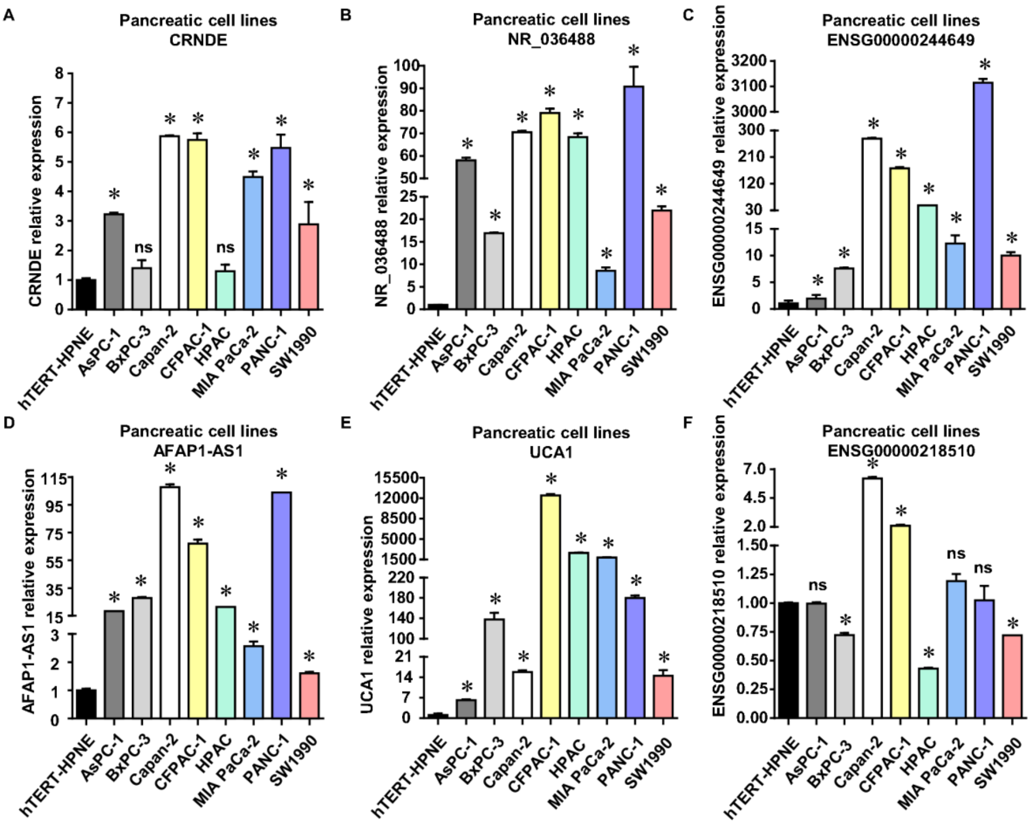

Supplementary Figure legends

Figure S1. Validation of candidate lncRNAs in PDAC cell lines by qRT-PCR analysis.

(A-F) Relative expression level of CRNDE, NR_036488, ENSG00000244649, AFAP1-AS1, UCA1 and ENSG00000218510 in 8 PDAC cell lines compared to the nonmalignant hTERT-HPNE cell line. *P<0.05 (Student's t-test), ns: not significant. The qRT-PCR expression data were all shown as mean±SD and normalised by GAPDH. GAPDH: glyceraldehyde-phosphate dehydrogenase; NP: normal pancreas.

Table S2A. siRNA sequences of UCA1 and ENSG00000218510 lncRNAs and nonspecific siRNA negative controls (NC)

Gene symbol	Sense	Antisense
UCA1	5'-GGGCUUGGGACAUUUCACUTT-3'	5'-AGUGAAAUGUCCCAAGCCCTT-3'
ENSG00000218510	5'-CCCAGAGACACAAAGAAAUTT-3'	5'-AUUUCUUUGUGUCUCUGGGTT-3'
NC	5'-UUCUCCGAACGUGUCACGUdTdT-3'	5'-ACGUGACACGUUCGGAGAAdTdT-3'

Table S2B. Primer sequences of six candidate lncRNAs and GAPDH reference gene

Gene symbol	Forward primer	Reverse primer
CRNDE	5'-TCAGCCGTTGGTCTTTGA-3'	5'-AACCTTCTTCTGCGTGACAA-3'
NR_036488	5'-CCGTGTAAAGAGGCCAGTGT-3'	5'-ACACGAGCCTTCACCATCAG-3'
ENSG00000244649	5'-GGGATTGGGTCCTGAGAAG-3'	5'-CACATTGAGGGAGCAGAGTG-3'
AFAP1-AS1	5'-TCGCTCAATGGAGTGACGGCA-3'	5'-CGGCTGAGACCGCTGAGAACTT-3'
UCA1	5'-CTCTCCATTGGGTTCACCATTC-3'	5'-GCGGCAGGTCTTAAGAGATGAG-3'
ENSG00000218510	5'-ATCCACACAAAGGAGCAGAC-3'	5'-CTGAGCAAGAAGCAAGCAG-3'
GAPDH	5'-GCATTGCCCTCAACGACCAC-3'	5'-CCACCACCCTGTTGCTGTAG-3'
